# Night-time confinement and the practice of realistic medicine

**DOI:** 10.1192/bjb.2018.83

**Published:** 2019-02

**Authors:** Lindsay Thomson

**Affiliations:** 1 University of Edinburgh, UK; 2 The State Hospital, UK; 3 Forensic Mental Health Services Managed Care Network, UK.

**Keywords:** Night-time confinement, realistic medicine, mental health legislation principles, cost effectiveness

## Abstract

**Summary:**

Night-time confinement is the practice of routinely locking patients in their rooms at night unless there is a contrary clinical indication. It is used in high-secure psychiatric hospitals. This article argues in favour of this practice on the basis of realistic medicine, an individual human rights based approach, the principles of mental health legislation in Scotland and England and cost effectiveness. This is not an academic debate. There is a real danger that those advocating against night-time confinement, if successful, will at best make little difference to the lives of our patients as they sleep, and at worst may hugely impoverish their lives because of reduced daytime activities.

**Declaration of interest:**

L.T. is Medical Director at The State Hospital. Night-time confinement is used within this setting.

I am not in favour of night-time confinement (NTC), but I am in favour of maximising the benefit to patients, and of efficiency and equity, as set out in the principles of mental health legislation throughout the UK; and I am for optimising our patients' opportunities for care, treatment and rehabilitation, and for ‘realistic medicine’.^1^ Realistic medicine places the patient at the centre of decision-making, and aims to reduce harm and waste, tackle unwarranted variation in care, manage clinical risk and innovate to improve so as to ensure a well-functioning and sustainable National Health Service (NHS). I am therefore not against NTC, the practice of routinely locking patients in their rooms at night unless there is a contrary clinical indication. This approach to NTC is used within the high-secure hospital (The State Hospital, Carstairs, Scotland) where I work.

The State Hospital is the high-secure psychiatric hospital for Scotland and Northern Ireland. In one capacity or another, I have worked there intermittently since 1992. Throughout this period we have always utilised NTC, but it is important to note that whether then or now, all patients who have need of an open door at night will have this; for example, because of poor mental or physical health, distress over a recent or pending event or risk of self-harm.

The European Committee for the Prevention of Torture and Inhuman or Degrading Treatment or Punishment (CPT) visited the State Hospital in 2003. It highlighted the poor accommodation for patients but the issue of NTC was never raised, although it was raised at Rampton Hospital in 1994. In September 2011, we moved to a purpose-built new hospital within the same site. Our previous estate could accommodate up to 250 patients within ten wards. In our old hospital some patients had an open door. This literally meant an open door. There was no door-locking mechanism for patients that could be overridden by staff. An unofficial quota operated within each ward. The wards contained between 24 and 26 patients and at any one time, approximately ten patients could have an open door. The admissions ward was excluded from this. Some patients did not want an open door because of their paranoia or legitimate concerns that other patients would enter their room. Others enjoyed the use of an open door, particularly as the majority of wards in the hospital at that time did not have en-suite facilities. This meant that patients could go to the toilet without having to ring a bell to attract the attention of staff.

The new hospital consists of four hubs, each with three wards for up to 12 patients. All rooms have en-suite facilities and televisions. The standard of these facilities is excellent, in fact, so good that a recent delegation from China asked to book in. The clinical model developed for the new hospital included the use of NTC for all patients. I emphasise again that any patient with a clinical need for an open door will have this. This allows us to use staff therapeutically during the day when patients are awake, rather than requiring an increased number of staff at night. Patients, named persons and carers were fully involved in developing the clinical model, during which time the views of these groups were considered, in addition to those of staff. Stakeholders reflected on the benefits of having staff available on shift during the day to support access to the grounds for escorted walks and to engage in structured activity.

NTC was also proposed because of the development of the en-suite facilities, the entertainment systems available in rooms and because the new hospital would be smoke free. No longer were the major motivating factors for an open door (the ability to go independently to the toilet and to get up to have a cigarette) relevant. NTC in its current form has been in place for almost 7 years. Never has this been the subject of complaint by a patient, named person or carer, nor raised by the Patient Partnership Group, the Carers' Group, the Independent Advocacy Service or the Mental Welfare Commission (MWC). The MWC is tasked under the Mental Health (Care and Treatment) (Scotland) Act 2003^2^ with visiting people, monitoring the legislation, influencing and challenging all those involved in mental health and protecting the human rights of those under the care of mental health services.

The State Hospital is not alone in its use of NTC. It is used in all four high-secure hospitals in the UK. Rampton Hospital^3^ evaluated its use of NTC and found that there was minimal change to the experience of patients and staff following the introduction of NTC, and that NTC did not affect the patients' quality of life or produce adverse effects; indeed incidents of self-harm and aggression, and hours in seclusion reduced during the evaluation. In addition, patients appeared to go to bed earlier and sleep better, and were therefore better able to utilise the therapeutic programme during the day. Research with similar findings was also carried out at Ashworth Hospital.^4^

In practical terms, removal of NTC within The State Hospital would potentially have major resource implications in terms of nurse staffing and costs. Currently at night (21.00–07.40 h) within The State Hospital, there are 20 nurses for ten wards, with three additional staff members floating to give extra cover as required, plus the senior nurse in charge. This is a total of 24 staff members. There are two ‘sides’ to the shift, so a minimum of 48 staff are required to ensure daily cover.

The model of nursing that would allow the safe opening of all bedroom doors would require a minimum of 39 nursing staff plus the senior nurse in charge. This is based on three staff per ward plus eight incident responders and a duty resuscitation nurse. This a total of 40 nurses, or 16 per night in excess of the NTC model.

To realise the projected staffing model that could support the delivery of a non-NTC model, significant additional staffing would be required to be allocated for night duty. As 16 additional staff would be required for each side of the shift, this equates to 32 staff per week. Forty additional staff would actually be required when the percentage allowance is added on for training, leave and other absences.

There would be two options to realise this potential requirement to increase staffing. One would be to reallocate staffing resources from day duty to night duty, thus reducing staff availability during daytime hours by 16 staff per day (or 1.6 staff per ward). This would have an additional staffing cost for the night shift of £373 000. The other option would be to employ 40 new nursing staff to bridge the gap that this model would create.

It would be exceptionally challenging to recruit this number of staff in one cohort, and there would be a potentially destabilising effect of having a large influx of new clinical staff into a high-secure environment. In financial terms, employing 40 new nursing staff would cost £1.6 million, based on a projected average cost of £40 000 per post. Given that in 2017–2018 the State Hospitals Board for Scotland balanced its books with little to spare, and that it is most unlikely that further funding would be forthcoming for this from Scottish Government, this could only be paid for by reducing daytime staffing. This would likely have a direct and immediate detrimental effect on the care and treatment of patients and is at odds with maximising the use of our staffing resource to best achieve safe, effective and person-centred care. As Scotland is moving to enshrine safe and effective staffing as part of our legislative framework, the current model of NTC could be argued to support achieving this legislative requirement in ensuring that our nursing staff are best deployed to meet the needs of our patients.

Undoubtedly some of the arguments above are utilitarian, but this in itself is an ethical theory. What then of other ethical and legal perspectives? The CPT report (2017)^5^ criticised the practice of NTC in English high-secure hospitals. It also criticised long-term segregation, but this is not a practice in use in Scotland. Specific powers to authorise NTC are set out in NHS England Security Directions (2013)^6^ and the 2015 revised Mental Health Act Code of Conduct.^7^ These state that NTC ‘should only be put in place where it is considered that this will maximise the therapeutic benefit for patients as a whole in the hospital’. CPT highlighted the importance of an individual perspective in the provision of psychiatric care and I would argue that each of our patients within The State Hospital is assessed for any negative effect that NTC may cause, and care is modified if this is identified. Indeed, The State Hospital adopted a human rights based approach over 10 years ago and this was independently evaluated by the Scottish Human Rights Commission^8^ and declared a good example for other public bodies. The UK Government^9^ in its response highlighted the public consultation on NTC, including patients in a high-secure setting, and the monitoring arrangements in place through the National Oversight Group for High Secure Services and announced its intention to review the use of NTC in light of the CPT's comments.

It is the view of the Royal College of Psychiatrists' Special Committee on Human Rights (SCHR) that NTC cannot be justified on ethical or human rights grounds (G. Szmukler, personal communication, 2017). The SCHR argue that NTC is incompatible with Article 5 (liberty and security) of the European Convention of Human Rights and probably Article 8 (private and family life), in that it is out of keeping with least restrictive measures found in the Mental Health Act Code of Practice 2015. SCHR considers the blanket imposition of NTC as an arbitrary restriction on liberty. They argue that NTC crosses a ‘red line’. Within The State Hospital, NTC was never introduced as a means to save money. It was brought in as part of a new clinical model that employed the same number of nursing staff for a reduced population, from 250 to 140 patients, but in smaller, more homely ward settings. Given that NTC within The State Hospital can be individually tailored regarding clinical needs and has never been the subject of any complaints or comments from patients, named persons, carers, advocacy, the Patient Partnership Group, staff or the MWC, it seems extreme to suggest it crosses a red line or that it constitutes inhuman or degrading treatment.

The SCHR suggests that NTC would be unlikely to meet criteria for a lawful restriction of persons' residual liberty according to *Munjaz v UK* 2913/06 [2012] ECHR 1704. Residual liberty refers to a further deprivation of an individual's liberty under Article 5 of the European Convention of Human Rights even if you are already detained. Further, they argue that Article 8 of the European Convention of Human Rights strengthens the importance of considering further restrictions in someone already detained, whose personal autonomy is limited.

SCHR argues that NTC is not compatible with the principles of the 2015 revised Mental Health Act Code of Conduct in England, chiefly the least restrictive option and maximising independence; respect and dignity; and purpose and effectiveness. The 2015 revised Mental Health Act Code of Conduct sets out five principles: least restrictive option and maximising independence; empowerment and involvement; respect and dignity; purpose and effectiveness; and efficiency and effectiveness. All principles are of equal importance, but their weighting may change depending on the context and nature of the decision being made. Surely here it is the principles of efficiency and equity, and purpose and effectiveness in promoting a system that supports recovery, that are of more importance than the least restrictive alternative. The Mental Health (Care and Treatment) (Scotland) Act 2003 has the underlying principle of least restrictive alternative enshrined within Section 1. It states that any function of mental health legislation should be discharged in a manner that appears to involve the minimum restriction on the freedom of the patient that is necessary in the circumstances. The person who is discharging the function shall have regards to the importance of the provision of appropriate services to the person who is subject to the certificate or the order concerned. It can be argued that minimum restriction should involve an open door at night-time, but this may be at the expense of providing appropriate services. Another principle contained within the Act is that of ‘the importance of providing the maximum benefit to the patient’. It is surely more important to have therapeutic opportunities open to the patients during their waking hours and to fulfil the principle of maximum benefit rather than argue that this is superseded by the principle of least restriction.

The debate about NTC is an example of the choices that the NHS, public, health professions and UK Government face. A legal case may result in a decision that removes NTC as an option, but this does not remove our responsibility for such a decision if we promoted this to the CPT and we promote legal arguments in favour of removing NTC. Civil court decisions are seldom based on right or wrong. They reflect and may lead societal thinking. So have those who advocate for the abolition of NTC got this right and are thinking in advance of those of us mired in, or knowledgeable about, the practicalities of running high-secure services? Or are they advocating a view that, if successful, will at best make little difference to the lives of our patients as they sleep, given that we already open the doors of those in distress, and at worst may impoverish their lives because of reduced daytime activities if no new funding is forthcoming for the greatly increased costs? And even if new funding is made available, we have a responsibility to consider where it has come from in terms of NHS funding and what the opportunity costs will be. In considering the concept of value, increased daytime staffing is of high and personalised value to the patients, and increased night-time staffing is of low allocative (population) value. The CPT report highlights poor levels of activities for patients in high-secure care. At The State Hospital, this is our clinical priority. Patient activity levels will deteriorate if NTC is no longer permitted. I firmly believe that the principles of maximum benefit and efficiency and equity outweigh least restrictive alternative in the case of NTC. Indeed, removal of NTC may increase restrictions on patients during the daytime. NTC should remain in place. Improved opportunities for patient activity should be the campaign that unites us all.

## About the author

**Lindsay Thomson** is professor of forensic psychiatry at University of Edinburgh; medical director at The State Hospital, Carstairs; and director of Forensic Mental Health Services Managed Care Network, UK.
